# Metallic glass coating for improved needle tattooing performance in reducing trauma: analysis on porcine and pig skins

**DOI:** 10.1038/s41598-020-77341-2

**Published:** 2020-11-23

**Authors:** Jinn P. Chu, Wen-Che Liao, Pakman Yiu, Ming-Tang Chiou, Kuan-Hsuan Su

**Affiliations:** 1 Department of Materials Science and Engineering, National Taiwan University of Science and Technology, Taipei, 10607 Taiwan, ROC; 2 Applied Research Center for Thin-Film Metallic Glass, National Taiwan University of Science and Technology, Taipei, 10607 Taiwan, ROC; 3 Department of Veterinary Medicine, National Pingtung University of Science and Technology, Neipu, Pingtung 91201 Taiwan, ROC

**Keywords:** Skin manifestations, Materials for devices, Nanoscale materials

## Abstract

The dissemination of tattooing into mainstream culture has raised concerns pertaining to the medical implications of these practices. This paper reports on the coating of tattoo needles with metallic-glass (MG) to reduce trauma to the skin. Extensive experimentation using pork samples and live pigs demonstrated the beneficial effects of non-stick MG coatings. Following 30 insertions into pork skin, significantly less tissue adhered to the MG-coated needles than to uncoated needles. MG-coated needles were also shown to reduce the spread of pigment to the surface of surrounding skin by up to 57%. This resulted in narrower tattoo lines of higher density, indicating that MG-coated needles could be useful in high-resolution tattooing. Histopathological analysis on live pigs revealed severe trauma induced by bare needles, as indicated by the secretion of fluids immediately after tattooing. The wounds formed by coated needles closed within 2 h after tattooing; however, those formed by bare needles remained open for at least 2 h and inflammation was still observed after 3 days. At 5 days after tattooing, skin punctured by the coated needle was entirely healed, whereas skin punctured by the bare needle was still covered with scabs. In addition to the medical benefits, it appears that MG-coated needles could improve the quality of tattoos, based on the fact that the amount of pigment retained in the skin is inversely proportional to the trauma caused by needles.

## Introduction

Tattooing is a permanent make-up process in which a set of needles is used to insert pigment into the dermal skin layer, which is approximately 2-mm thick. In most countries, tattooing is not regulated as a medical procedure, despite the fact that it is a type of minor surgery in which the skin is punctured thousands of times. Complications (e.g., infection) are not uncommon and often require medical treatment^1–3^. Numerous case reports describe tattoo-associated clinical issues attributed to pigments (e.g., allergic reactions)^4,5^ as well as tattoo needles (e.g., needle-induced trauma)^6^. In extreme cases, needle trauma presents as an open wound or traumatic excoriation requiring medical intervention and extended healing. Pigment overload is another common complication, which can lead to chronic medical issues (e.g., granulomatous inflammation induced by pigment agglomeration) after the tattoo has healed^6^.

The term MG (metallic-glass) refers to a specific group of amorphous multi-component metallic alloys. MG coatings feature high strength, good ductility, a smooth surface, low friction, and high hydrophobicity, exceeding those of other alloys and ceramic coatings^7,8^. The non-stick characteristics of hydrophobic materials make them ideal for medical devices, such as needles, scalpels, microtomes, dermatomes, and guide wires^8–12^. In previous studies, we compared the performance of different kinds of coatings^9,10^ and demonstrated that MG coatings can reduce insertion/retraction resistance into porcine tissue and rubber by as much as 60–70%^9^. Compared to bare needles, MG-treated needles have also been shown to reduce the area of needle-induced trauma by up to 44%^11^.

In this study, we coated tattoo needles with metallic-glass (MG) to reduce needle-induced trauma, accelerate healing, and improve tattooing performance in terms of pigment retention in the tattooed skin. Extensive experiments were conducted to compare bare and coated needles using pork skin (up to 30 manual insertions) and live pigs (hundreds of insertions using a tattoo gun). Zr-based MG system was evaluated in terms of hydrophobicity. Using seven common color pigments, it was found that MG-coated needles reduced the spread of excess pigment across tattoos on pork skin by as much as 57% (compared to bare needles), while improving the retention of pigment in the skin. MG-coated needles were also shown to reduce the severity of needle-induced trauma and accelerate healing. Our results clearly demonstrate the potential of MG-coated needles in improving tattooing performance.

## Experimental methods

The 316 stainless-steel tattoo needles used in this study were #12-gauge (diameter = 350 µm) round-liner needles (Aerolite, BELLEZA TATTOO AGENCY). We evaluated two needle configurations (1205RL and 1209RL), respectively comprising 5 needles and 9 needles in a round grouping (hereafter referred to as 5-needle and 9-needle). The MG coatings were deposited on the needles using a high-power impulse magnetron sputtering system (HiPIMS, Highpulse Bipolar 4002 G2, TRUMPF Hüttinger) under vacuum with base pressure of < 9.3 × 10^–4^ Pa and working pressure of 5.0 × 10^–1^ Pa. During deposition, the power range was 1–2.5 kW and the working distance was maintained at 8.68 cm from the 6-inch alloy targets to achieve a deposition rate of 7.1 nm/min. The needles were placed on a planetary-rotation turntable to ensure coatings of uniform thickness. The nominal coating thickness was 270 nm. Using an electron probe micro-analyzer, the composition of the MG coating (in atomic percentage) was estimated as follows: Zr: 62.4%, Cu: 22.4%, Al: 9.8%, and Ni: 5.4%.

The hydrophobicity of the MG coatings was evaluated using contact angle measurements (Phoenix, SEO) of seven color tattoo pigments (black, red, blue, green, orange, purple, and yellow) on sheets of 316 stainless steel. The black pigment was obtained from DYNAMIC COLOR Co. and the other colors were obtained from RADIANT COLORS Co. Black, blue, and red pigments were used in the tattooing of pig skin and pork samples. The tattooing of pig skin was performed using a set of tattoo needles mounted on a coil-driven tattoo machine (Sidewinder 7, DK ROTARY) with a stroke length of 1.5–3 mm operated at 50–200 Hz by a professional tattoo artist. The MG coating was characterized for its crystallographic structure by an X-ray diffractometer (XRD, BRUKER D8 DISCOVER) with monochromatic Cu Kα radiation. Dual-beam field-emission focused ion beam (FIB) system (Quanta 3D FEG, FEI) operated in scanning electron microscopy (SEM) mode was used for the characterization of needle surfaces. FIB sectioning of the coated needle enabled observation of the coatings in cross section before and after tattooing.

Non-animal testing was performed on pieces of pork skin purchased from a supermarket following inspection by the Certified Agricultural Standards (CAS) of Taiwan. Experiments involving needle insertion into pork skin were performed at a speed of 20 mm per second to a depth of 3 mm using a material testing system (Criterion 42.503 Test System, MTS). A laser confocal microscope (OLS5000, OLYMPUS) was used to examine the surface topography of the pork skin. ImageJ imaging software was used to quantify the distribution of pigment on the pork skin. Following insertions into the pork skin, the needles were examined using a fluorescent microscope (DM2000, LEICA) with the aid of a blue fluorescent nucleic acid stain, DAPI (4′,6-diamidino-2-phenylindole, Dihydrochloride) to reveal the attachment of biological material.

Pigs subjected to animal testing were treated in strict accordance with protocols approved by the Institutional Animal Care Committee of National Pingtung University of Science and Technology, based on the IACUC protocol for animal care (approval No. NPUST-109-001). Six ten-week-old pigs (each weighing approximately 30 kg) were used in the experiments. The pigs were anesthetized using ZOLETIL (15 mg/kg, intramuscular injection) during the tattooing sessions, which lasted approximately 2 h. Tattooing was performed on the ventral skin over an area of ~ 25 cm^2^. The tattooed pigs were then examined after 2 h, 6 h, 1 day, 2 days, 3 days, and 5 days. The tattooed pigs were subjected to necropsy after sacrifice, and tissues from the epidermal layer to the subcutis layer of the black tattoo region were collected, fixed, sliced to 4-µm thickness, and stained with haematoxylin and eosin. These histopathological lesions were then examined under an optical microscope (DP71, OLYMPUS).

## Results and discussion

**Figure 1:**
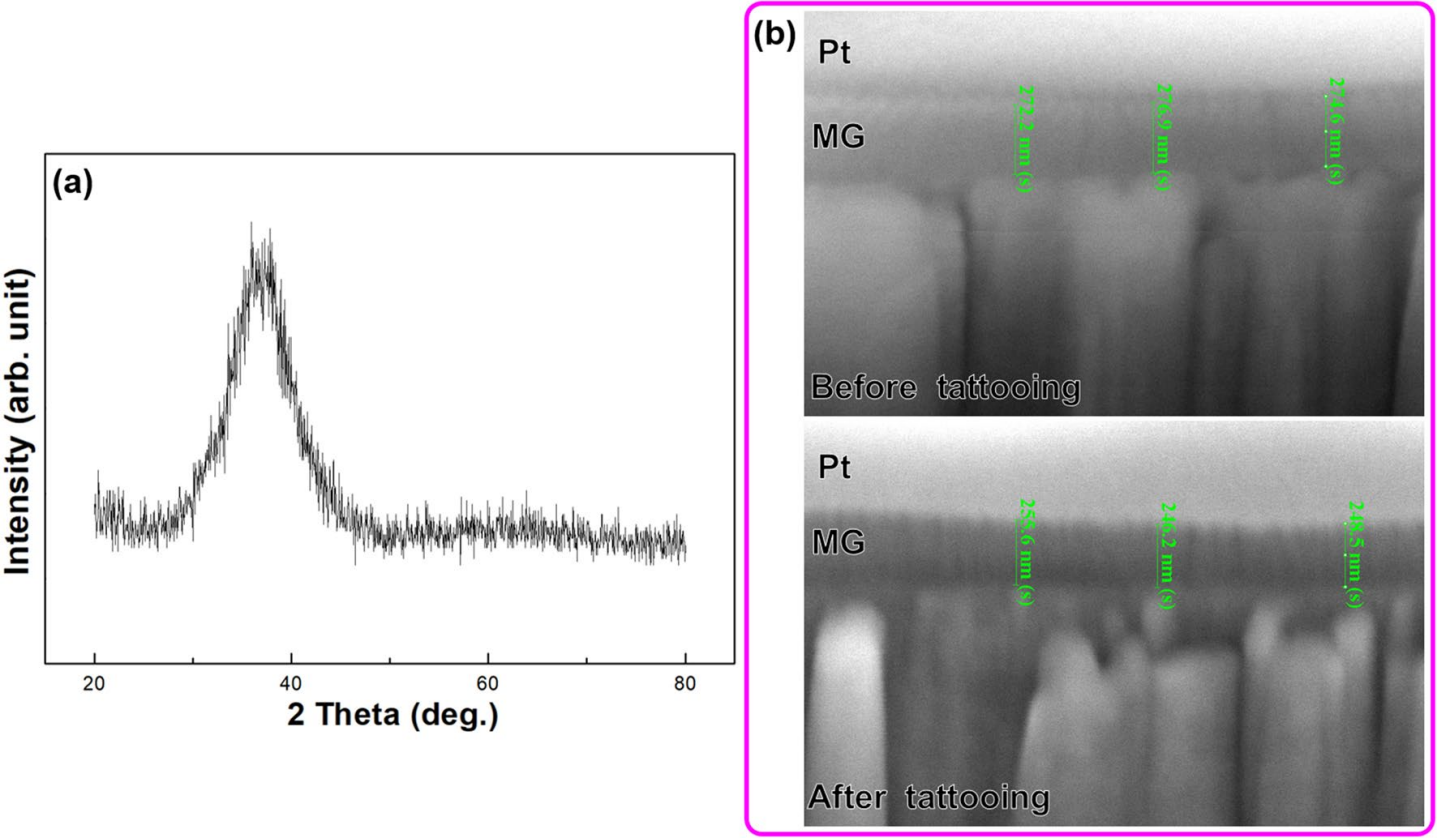
(**a**) XRD crystallographic analysis result of MG coating. (**b**) Cross-sectional SEM micrographs of coated needles before and after tattooing for 25 cm^2^ area on a live pig. Cross-sectional samples were sectioned by FIB. Pt as the protective layer was deposited during FIB cutting.

In a previous study^8,9^, Zr-based MG was shown to be highly hydrophobic, based on water contact angle measurements. In the current study, water was used as a solvent for the tattoo pigment; therefore, we anticipated that the results would be similar. For the sake of comparison, Al-based MG and W-based MG were included for hydrophobicity evaluations. As shown in Table S1 in Supplementary Information, the Zr-based MG presented a contact angle higher than that of Al-based MG and W-based MG, regardless of the color pigment. This means that the adhesion of pigment to Zr-based MG was weaker than the adhesion to Al-based MG and W-based MG coatings as well as the bare stainless steel. We therefore selected Zr-based MG as the preferred coating for subsequent experiments. As described later, the excellent hydrophobicity of the Zr-based MG coating proved highly beneficial in reducing excess pigment and skin trauma. Crystallographic analysis based on XRD shown in Fig. 1a reveals the hump between 25 and 45 degrees of 2 theta in the pattern, confirming the amorphous nature of Zr-based MG coating. Focused ion beam (FIB) characterization was used to evaluate the adhesion between the needle and the coating. Based on SEM cross-sectional images after FIB sectioning in Fig. 1b, the process of tattooing a live pig (area of 25 cm^2^) was shown to reduce the thickness of the coating from approximately 275 nm to ~ 250 nm. These results demonstrate that the adhesion between the coating and the needle was very good, based on the fact that the coating remained firmly attached even as the harsh conditions of tattooing wore down the coating.

### Part 1: Non-animal testing

**Figure 2:**
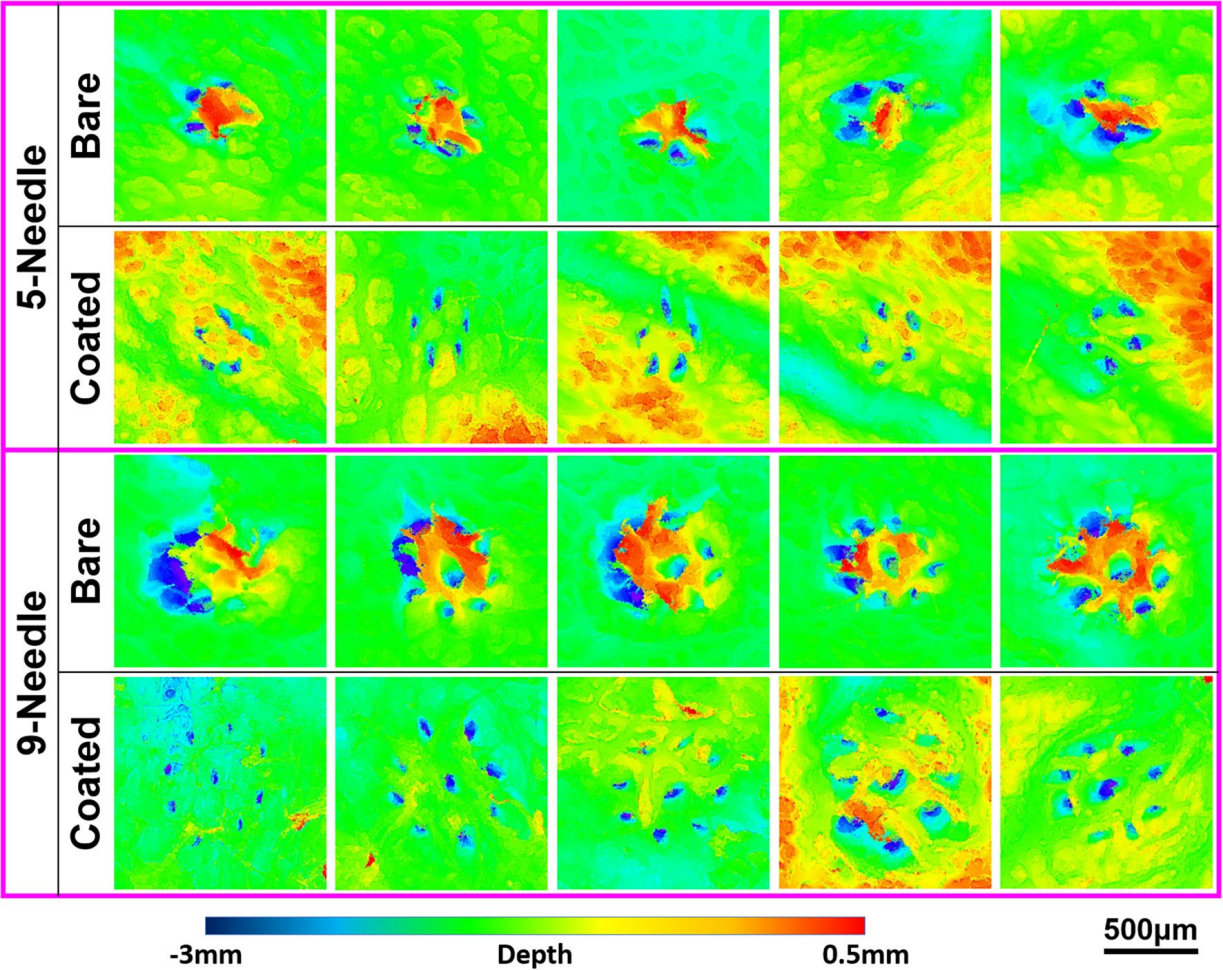
Confocal microscope images of pork skins following one-time insertion tests with bare and coated 5- and 9-needles. The images reveal the skin surface height from high to low by the red to blue color gradient, respectively.

Figure 2 presents confocal microscope images of pork skin samples following one-time insertion tests using bare needles and coated needles (5- and 9-needle sets). The red to blue color gradient indicates the skin surface height from high to low, respectively. In the middle of the tattoo pattern produced using bare needles, the skin was noticeably raised in accordance with the arrangement of the needles. This can be attributed to friction forces pulling the pork tissue during the retraction of the needles, as demonstrated in video segments in our previous study^9^. By contrast, the tattoo pattern produced using MG-coated needles remained smooth and flat after retraction, due to the reduced coefficient of friction. Note that the effects of needle retraction were far more pronounced in the 9-needle case than in the 5-needle case. Regardless of whether five or nine needles were used, all of the coated needles presented well-defined punctures.

**Figure 3:**
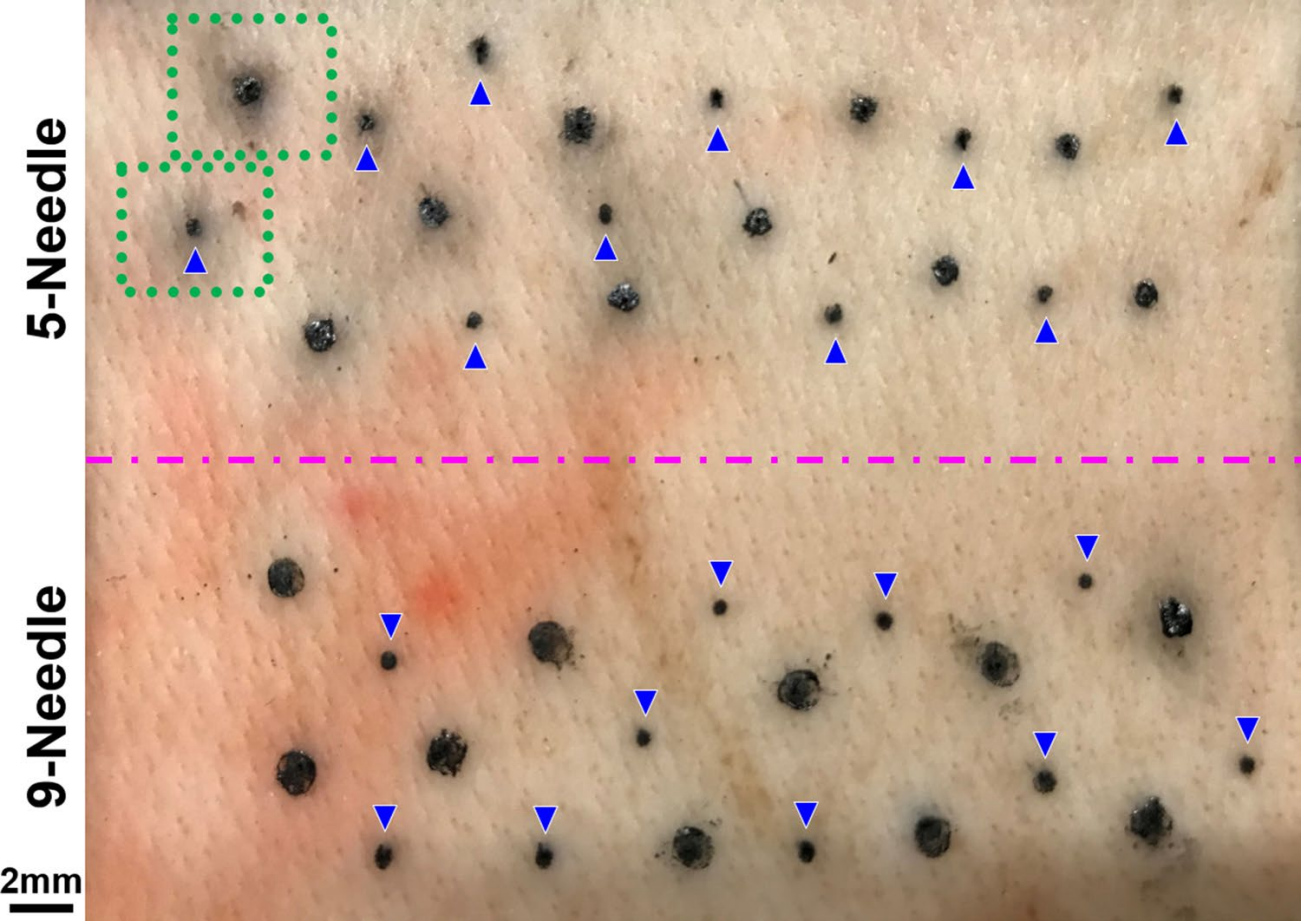
Photographic image of tattoos on pork skin created using bare and coated needles in random positions. Each tattoo consisted of 30-time insertion using the same set of needles. 10 sets of needles were used in each bare and coated conditions (40 sets of needles in total). Note that excess pigment was not removed from the surface between insertions. Small-sized tattoos marked by blue arrows were created by coated needles. Green dots outline the areas for measuring the pigment distribution demonstrated in Fig. 4.

**Figure 4:**
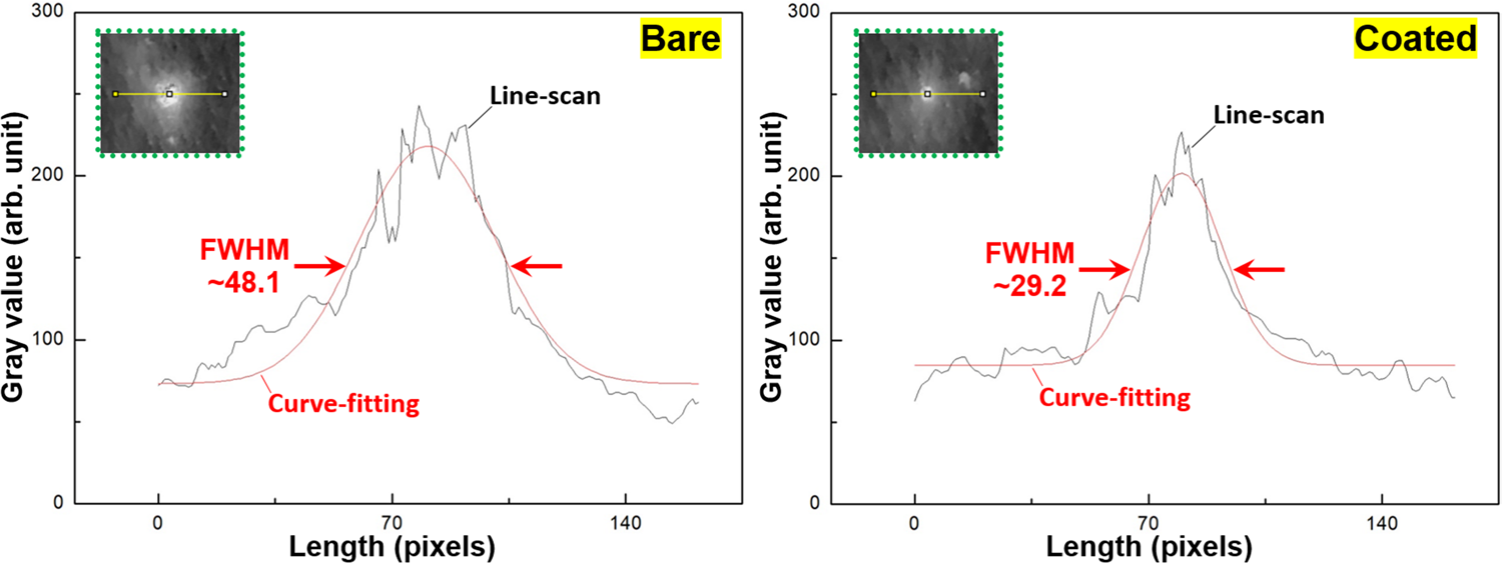
Line-scan gray-level pigment intensity profiles across tattoos created by bare and coated 5-needle using the profile plot tool of ImageJ software. A Gaussian function was used to fit the profile curve in red color and obtain the full width at half maximum (FWHM) pixel value of intensity profile. The profiles were obtained from areas as an example outlined by green dots shown in Fig. 3, with FWHM pixel values of 48.1 for bare and 29.2 for coated tattoos. 1 pixel is equal to ~ 25.6 µm.

Figure 3 presents a photographic image of tattoos on pork skin created using bare and coated needles in random positions. In this test, we used ten sets each of 5-needle and 9-needle arrays in bare and coated conditions (40 sets in total). Each tattoo was created using 30 insertions (using the MTS system) with the same set of needles. Note that excess pigment was not removed from the surface between insertions. The coated-needles did not leave as much residual pigment on the skin as did the bare-needles, which could conceivably be beneficial to creating high-resolution tattoo patterns and reduce pigment consumption. The full width at half maximum (FWHM) of intensity profiles was used to quantify the distribution of pigment in the tattoos. This involved converting images of tattoos (Fig. 3) to gray scale values in order to obtain line-scan intensity profiles of the tattoo pigments using the profile plot tool in ImageJ software. Note that the values were averaged from at least three measurements. As shown in Fig. 4, a Gaussian function was used to fit the profile curve and obtain the FWHM results for use in comparing tattoos created using bare and coated needles. As shown in Table 1, the MG coatings significantly reduced the FWHM pixel values: 5-needles (from 39.4 to 21.5; 45%), 9-needles (from 49.0 to 21.3; 57%). The broad spreading of pigment around tattoos created using the bare needle can presumably be attributed to skin damage, similar to that shown in Fig. 2. These results are in good agreement with previous reports on large-gauge (18G) single needles, in which coated needles were shown to reduce fracturing in pork skin samples by approximately 44%, compared to bare needles^11^.

**Table 1:** Average full width at half maximum (FWHM) pixel values of line-scan gray-level intensity profiles obtained from tattoos created by bare and coated 5- and 9-needles in the image of Fig. 3.

Type of needle	Bare	Coated
5-needles	39.4 ± 5.3	21.5 ± 3.0
9-needles	49.0 ± 3.5	21.3 ± 3.1

Note that at least three measurements were performed on each tattoo created by the needle. Each set of bare and coated 5- and 9-needles created 10 tattoos at various locations on the pork skin. 1 pixel is equal to ~ 25.6 µm.

**Figure 5:**
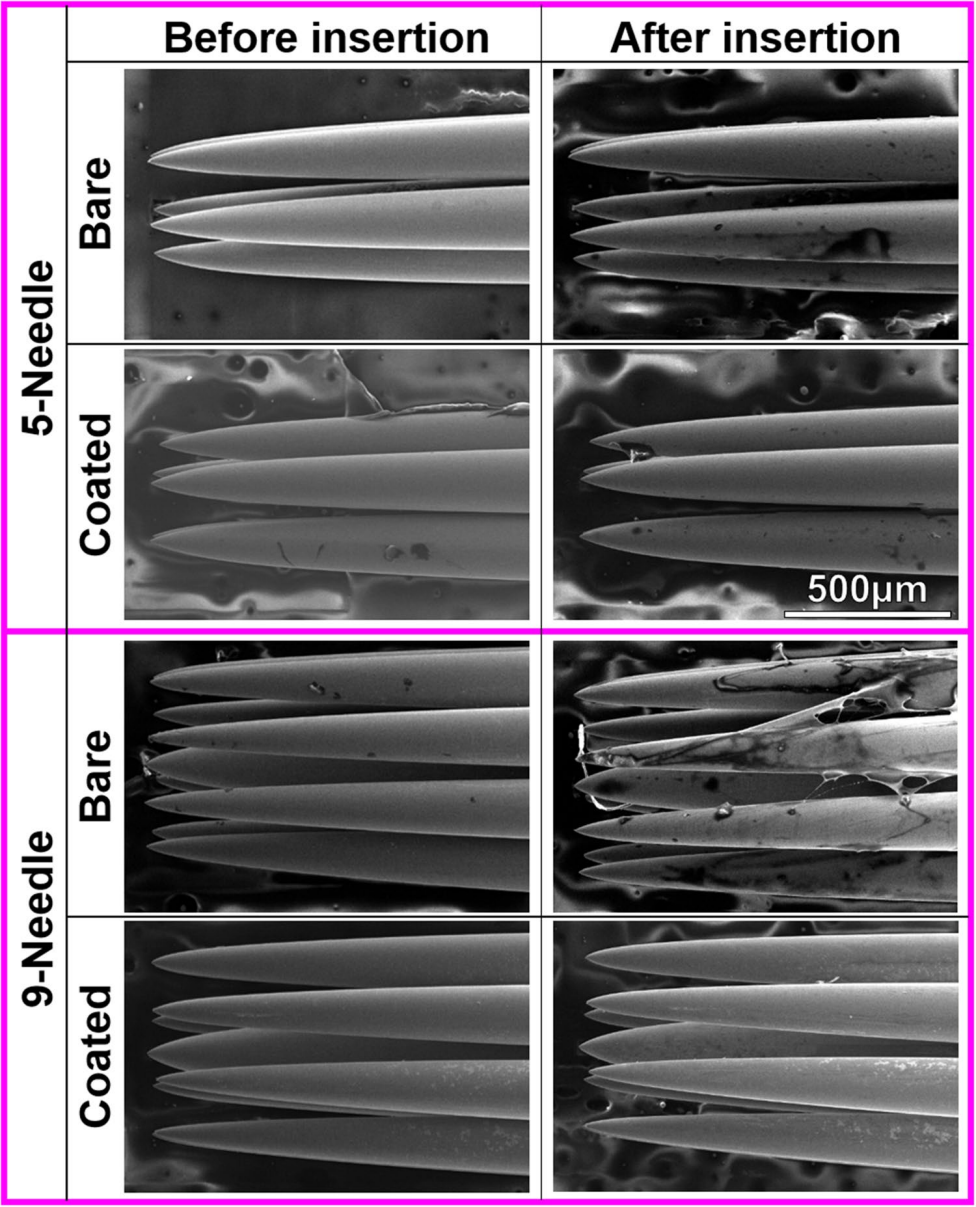
SEM images of bare and coated needles before and after 30-time insertion tests on the pork skin.

**Figure 6:**
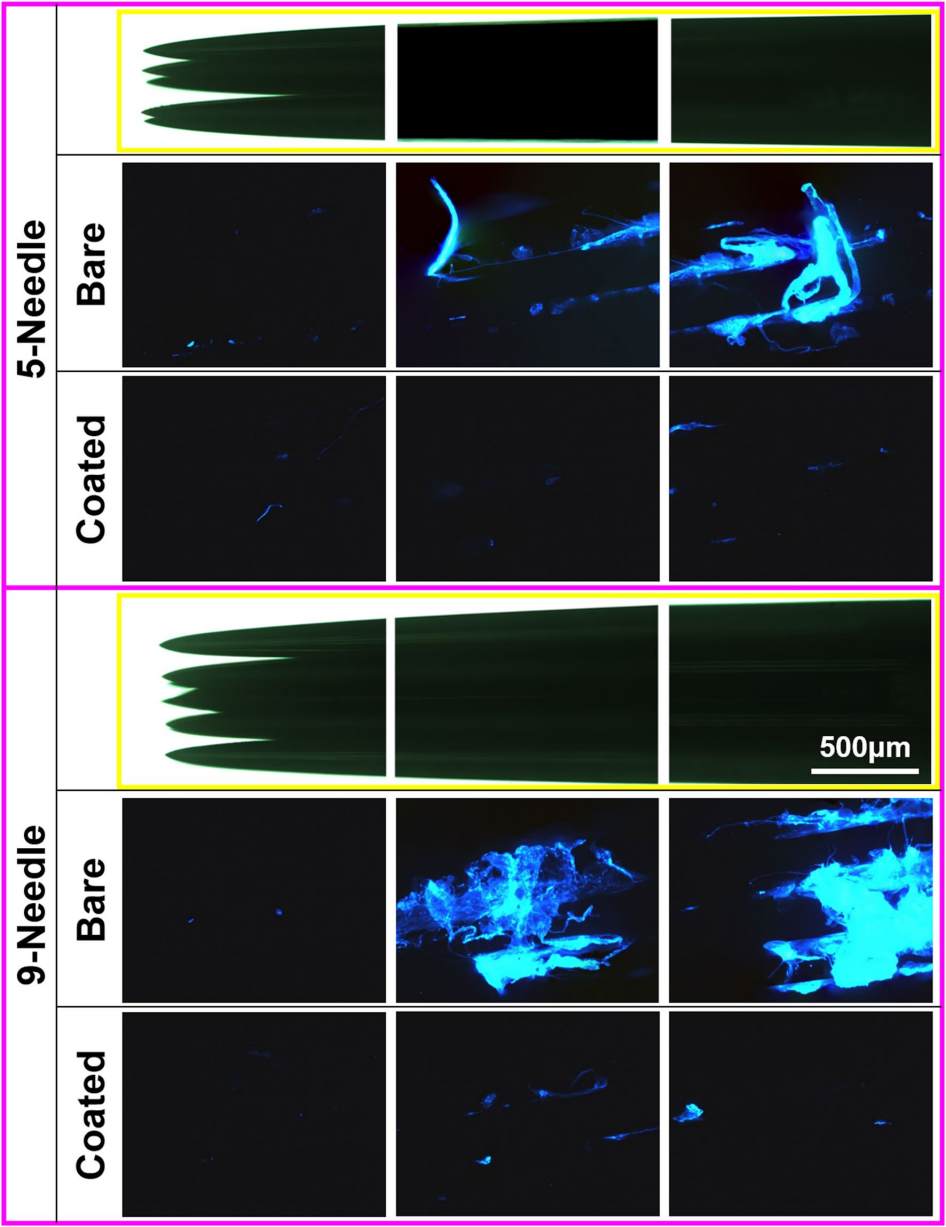
Fluorescent microscope images showing the area of cell remaining after 30-time insertion of bare and coated needles test on the pork skin. To facilitate the visual comparison, optical microscope images of needle parts selected for fluorescent observation are framed in yellow lines.

Following 30 insertions into the pork skin, the needles were evaluated using SEM, the results of which are presented in Fig. 5. We observed far more biological tissue on the surfaces of the bare needles than on the coated needles, regardless of the needle configuration (5- or 9-needles). The low atomic numbers of elements in tissue (e.g., C and O) causes tissue to appear dark in SEM images, whereas the metallic needles appear bright. Fluorescence microscopy images obtained from DAPI-stained needles (Fig. 6) clearly show the adhesion of tissue on the needles. These images also demonstrate the benefits of the non-stick MG coating in reducing tissue adhesion. Note that there was more porcine tissue on the 9-needle arrays than on the 5-needle arrays. We also observed more tissue in the posterior region of the needles than on the tips, due to the repeated insertion of the needles to a depth of approximately 3 mm, which effectively wiped the tips clean. It is reasonable to assume that tissue adhesion is proportional to the amount of damage inflicted on the pork skin by the friction of the needles. Thus, these findings support our previous observation that the MG coating reduced the stickiness (friction) of the needles. Overall, these results are consistent with those obtained from MG coated hair-transplant needles following 80 insertions into rubber samples^13^.

### Part 2: Animal testing

**Figure 7:**
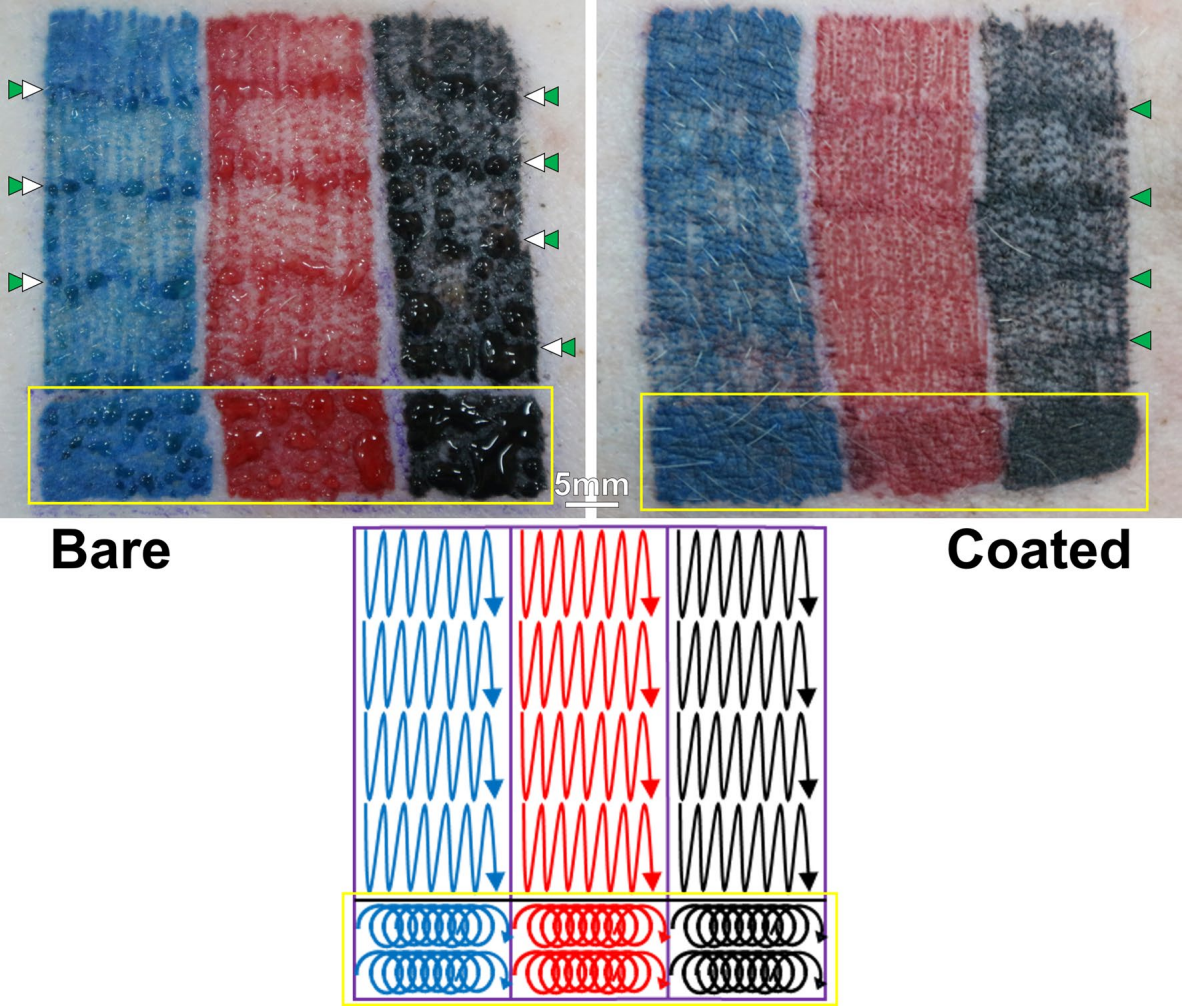
Photographic images of pig skins following tattooed in blue, red and black pigments by bare and coated 5-needle sets, with a schematic illustration of needle paths in the bottom. The tattoos were done in two needle path patterns: lines and circles. Yellow frames indicate the tattoos in the circle pattern. Excess pigments were removed before taking these photos. White arrows point out some examples of the presence of secretions, while green arrows indicate the turning points of line pattern.

The application of color is a crucial aspect of the tattooing process. Figure 7 presents photographs of typical tattoo samples on the skin of live pigs after removing excess pigment from the surface. In this study, the shading and filling of tattoos was performed using two needle path patterns: lines and circles. Clearly, the bare needles resulted in severe skin damage, which caused skin secretions even after excess pigment was removed (see the white arrows in this figure). We can assume that the severity of the secretions was proportional to the severity of skin damage. Thus, it appears that the most severe skin damage occurred in regions subjected to circular fill-in patterns (yellow frame) and at the points where lines turned (green arrows). This type of skin damage is a sign of overworked tattoos or “needle trauma”^6^. Unlike the bare needles, the MG-coated needles produced very little skin damage, as indicated by a lack of secretions from the skin. The coated needles also appear to have preserved the texture of the skin, even in regions that were filled in using a circular pattern.

Filling in a region of a tattoo requires that the needles trace multiple paths; however, the coloring often appears rich only in areas where those paths overlap. This effect is particularly evident in the regions of red color applied using the bare needle in Fig. 7. By contrast, the coloring applied using the coated needles appeared more continuous, due to the density of color in areas without overlapping lines. This color density can be attributed to the higher concentration of pigment in the small individual dots created by the coated needles. This is consistent with the small puncture marks observed in the pork skins (Figs. 2, 3). Thus, it appears that the non-stick characteristics of the MG-coated needles may be beneficial to the quality of the resulting tattoo. Furthermore, by minimizing the resistance of the needles^9,11^, the MG-coatings allow for smoother insertion/retraction motions, making the tattoo gun easier manipulate and reducing perturbations and discomfort in soft skin.

**Figure 8:**
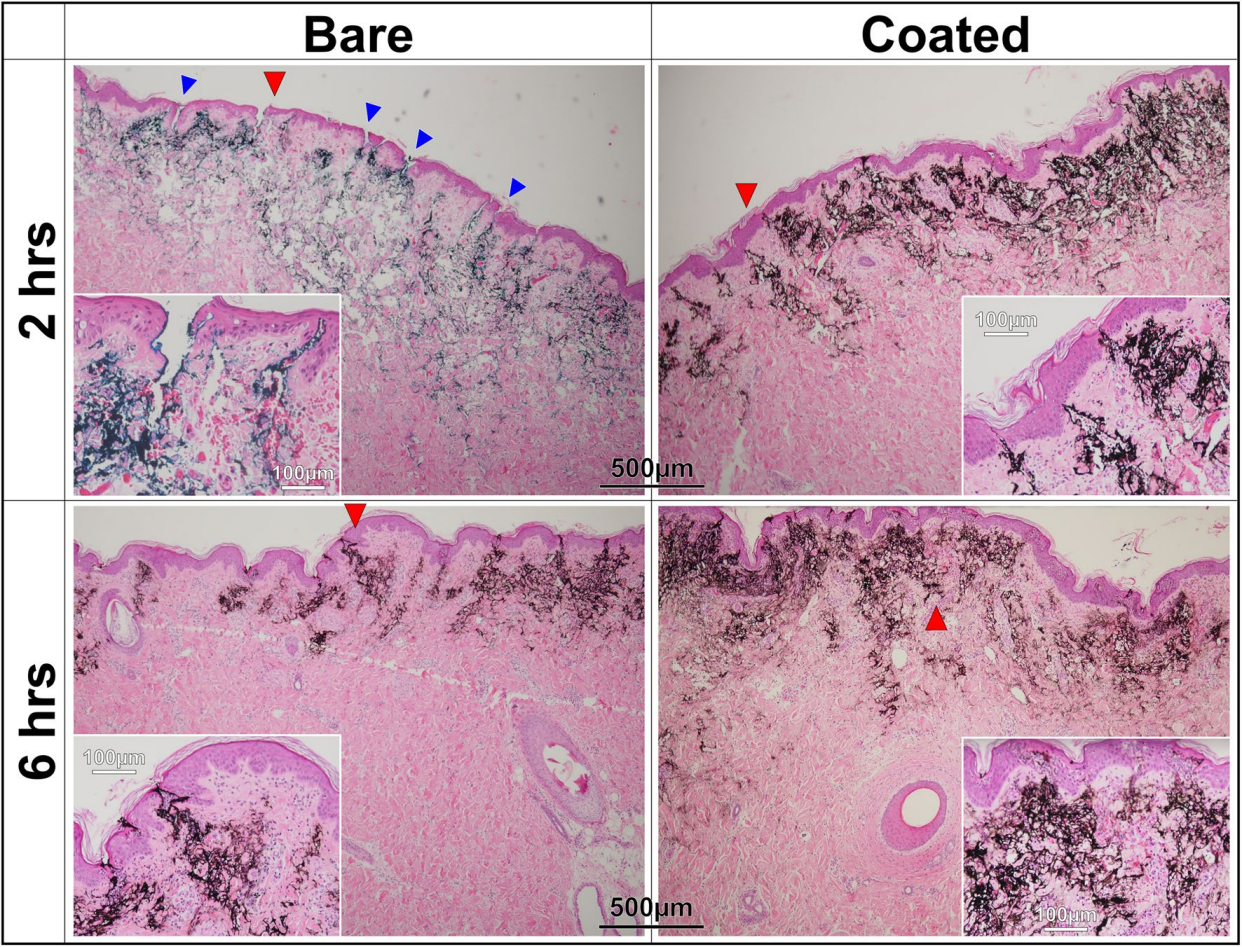
Histopathological images of pig skins taken after 2 and 6 h following tattooed in black pigment by bare and coated 5-needle sets. Blue arrows in 2-h images indicate some examples of open puncture wounds on the skin, while red arrows designate the areas for magnified views in their respective insets.

**Figure 9:**
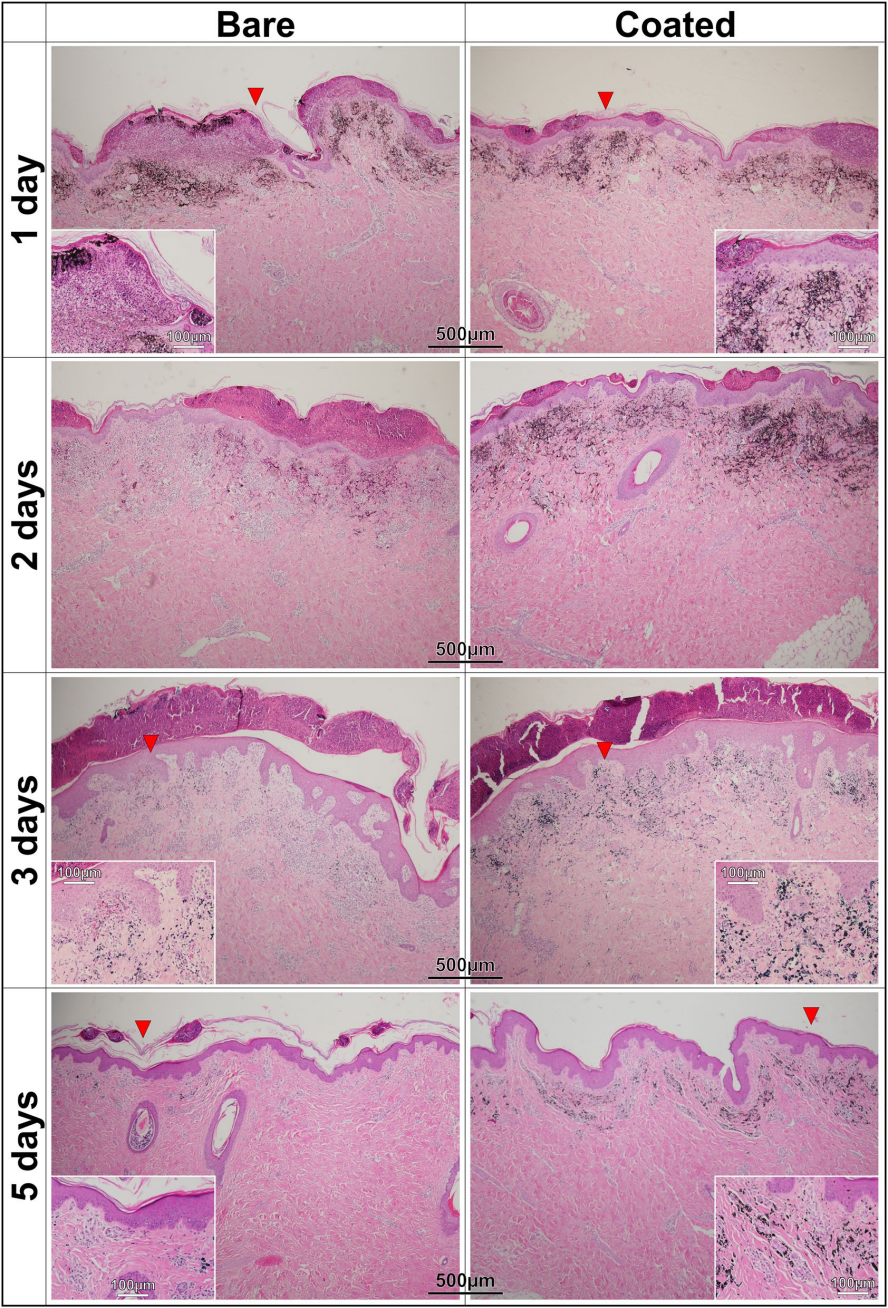
Histopathological images of pig skins taken after 1, 2, 3 and 5 days following tattooed in black pigment by bare and coated 5-needle sets. Red arrows designate the areas for magnified views in their respective insets.

Figures 8 and 9 present the image analysis results of histopathological samples obtained at various intervals after the tattoo operation. Note that the samples obtained at each interval were from the same animals to minimize variability. The images in Fig. 8 clearly show signs of needle trauma induced by bare needles. At 2 h after tattooing, the tattoos created using bare needles showed numerous open puncture wounds (20–75 µm); however, the tattoos created using coated needles showed few signs of trauma. We can also see in Fig. 8 that the puncture wounds caused by the bare needles healed far more slowly (6 h) than did the small punctures caused by coated needles. Furthermore, the black pigment applied using bare needles was dispersed over a wider area. The images obtained at 6 h revealed far less pigment in the tattoos created using bare needles than in those created using coated needles. This discrepancy can presumably be attributed to a release of pigment with the skin secretions mentioned previously. The insets of Fig. 8 present magnified images obtained at 6 h after tattooing. These images show the infiltration of inflammatory cells (mainly neutrophils) accompanied by the accumulation of large quantities of necrotic cell debris. The wounds caused by bare needles showed infiltration by a large number of eosinophils into the epidermis and dermal papilla layer. The same situation was encountered with the coated needles; however, the epidermal areas affected by inflammation had better tissue coverage.

As shown in Fig. 9, on the first day after tattooing, many of the wounds caused by the bare needles showed signs of inflammation due to localized neutrophil infiltration. By contrast, the wounds caused by the coated needles had already begun to recover, as indicated by the formation of new skin. More black pigment appeared in the inflamed regions caused by bare needles than in those caused by coated needles.

On the second day after tattooing, we observed a clear boundary between the wounds and the normal epidermal layer, regardless of the type of needle that caused the wound. Nonetheless, the scabs on wounds caused by the coated needles had already begun peeling; *i.e.*, restoring normal skin conditions. Again, we observed a higher concentration of pigment in the coated needle tattoos than the bare needle tattoos.

On the third day, scabs on wounds created by the bare needle were starting to peel off, despite a large number of inflammatory cells (eosinophils) infiltrating the dermal papilla layer. We also observed necrotic cells and abnormal partial bleeding in the dermis of the tattooed skin. The bleeding can be seen in the inset image. Neither of these conditions were observed in the tattoos applied using coated needles.

By the fifth day, the wounds caused by the coated needles were fully healed, while many of the wounds caused by the bare needles were still covered with scabs. Much of pigment observed in regions of inflammation on day 1 had already been flushed from the skin during the healing process (Fig. 9).

Overall, the tattoos created using bare needles on six different pigs presented a continual decrease in pigment concentration over the five-day recovery period. In fact, by the fifth day, the tattoos had become nearly indistinguishable unless observed under high magnification (see the inset image). The histopathological images in Figs. 8 and 9 were subjected to further analysis using ImageJ software to determine the concentration of black pigment, the results of which are presented as pixel values in Table 2. At 2 h after tattooing, it was observed that the bare needle produced pigment concentrations that were slightly higher (~ 9%) than those produced using the coated needle. However, the pigment concentration in tattoos from the uncoated needle dropped by more than 60% within 6 h. It appears that this substantial decrease in concentration can be attributed to pigment being transported out by secretions resulting from severe skin trauma. By contrast, most of the pigment implanted by the coated needles (~ 94%) remained in the skin at 6 h. Throughout the recovery period (1 to 5 days), skin trauma and inflammation were again shown to play key roles in the retention of pigment. There have been reports of pigment being transported from tattooed skin to peripheral organs (e.g., lymph nodes) via the blood stream^14^. We thus expected that at least some of the pigment in regions of inflammation will eventually be flushed out of the skin (i.e., in the latter stages of healing), leading to a further reduction in pigment concentration. Obviously, a decrease in pigment concentration over time would be considered a poor outcome.

**Table 2:** Concentrations of black pigment as pixel values obtained from histopathological images of Figs. 8 and 9, using the ImageJ software.

Time	Bare	Coated
2 h	144,342	138,561
6 h	54,840	130,984
1 day	44,128	41,269
2 days	10,900	93,515
3 days	2,555	19,152
5 days	2,095	13,974

Note that 1 pixel is equal to ~ 1.92 µm.

## Conclusions

This study investigated application of an MG coating to tattoo needles. Extensive analysis revealed that the non-stick properties of MG significantly reduced the trauma induced by bare needles. Histopathological analysis on live pigs revealed severe trauma induced by bare needles, as indicated by the secretion of fluids immediately after tattooing. Furthermore, those wounds remained open for at least 2 h after tattooing with inflammation continuing for 3 days. The wounds formed by coated needles were far less severe, having closed within 2 h after tattooing. At 5 days after tattooing, skin punctured by the coated needle was entirely healed, whereas skin punctured by the bare needles was still covered with scabs.

Coating the needles with MG was also shown to reduce the spread of pigment into surrounding tissue by as much as 57%. This resulted in narrower tattoo lines of higher density, indicating that MG-coated needles could be useful in high-resolution tattooing.

## Supplementary information

Supplementary Information.
