# Supplementary material for: Metallic glass coating for improved needle tattooing performance in reducing trauma: analysis on porcine and pig skins
Source: Sci Rep. 2020 Nov 23;10:20318. doi: 10.1038/s41598-020-77341-2 (PMC7683722; doi:10.1038/s41598-020-77341-2)
Supplement: Supplementary file 1 — Supplementary Information. [file 41598_2020_77341_MOESM1_ESM.docx]

**<Supplementary Information>**

Metallic Glass Coating for Improved Needle Tattooing Performance in Reducing Trauma: Analysis on Porcine and Pig Skins

Jinn P. Chu, Wen-Che Liao, Pakman Yiu, Ming-Tang Chiou, Kuan-Hsuan Su

Table S1. Average contact angle values of various color pigments on bare and coated 316 stainless steel sheets with different MG systems.

| Type of pigment | Bare | Zr-based MG | W-based MG | Al-based MG |
| --- | --- | --- | --- | --- |
| Black | 13.9± 1.6 | 43.9 ± 1.0 | 31.2 ± 1.6 | 26.6 ± 1.2 |
| Red | 30.2 ± 0.6 | 52.9 ± 3.0 | 44.5 ± 1.0 | 33.3 ± 0.6 |
| Blue | 44.1 ± 1.1 | 64.1 ± 2.8 | 58.1 ± 0.9 | 52.6 ± 1.9 |
| Green | 25.3 ± 1.1 | 32.8 ± 0.7 | 30.9 ± 0.6 | 26.6 ± 0.4 |
| Orange | 32.2 ± 1.5 | 50.3 ± 0.8 | 39.1 ± 0.8 | 38.9 ± 0.9 |
| Purple | 26.6 ± 0.9 | 51.1 ± 1.5 | 32.9 ± 0.4 | 33.9 ± 0.4 |
| Yellow | 38.0 ± 1.1 | 61.2 ± 4.0 | 45.0 ± 0.7 | 34.7 ± 0.9 |

Note that each contact angle value was averaged from five measurements. Compositions of MGs are determined by EPMA as follows (in atomic percentage): W-based MG (W: 56.8%, Ni: 38.0% and B: 5.2%) and Al-based MG (Al: 96.0%, Y: 2.4%, and Ni: 1.6%)
